# Early injury-induced responses in the transected adult human sural nerve

**DOI:** 10.21203/rs.3.rs-9204646/v1

**Published:** 2026-06-18

**Authors:** Gabriela I. Aparicio, Jorge E. Quintero, Nataliya Timoshevskaya, Noelia D’Elia, Monica J. Chau, Nicholas Semack, Greg A. Gerhardt, Craig G. van Horne, Paula V. Monje

**Affiliations:** 1 Department of Neurosurgery, College of Medicine, University of Kentucky, Lexington, KY, USA.; 2 Department of Neuroscience, College of Medicine, University of Kentucky, Lexington, KY, USA.; 3 Neurorestoration Center; College of Medicine, University of Kentucky, Lexington, KY, USA.; 4 Spinal Cord and Brain Injury Research Center, College of Medicine, University of Kentucky, Lexington, KY, USA.; 5 Markey Cancer Center, College of Medicine, University of Kentucky, Lexington, KY, USA.; 6 Department of Biology, College of Arts and Sciences, University of Kentucky, Lexington, KY, USA.

**Keywords:** Human peripheral nerves, cell therapies, Schwann cells, clinical trials, Wallerian degeneration, axotomy

## Abstract

Performing a transection injury is a safe and practical method to potentially enhance the therapeutic value of transplantable nerve grafts. Our team has tested the autologous implantation of denervated fascicle pieces from a fully transected sural nerve as an experimental treatment for Parkinson’s disease. This study used nerve biospecimens from the clinical trial participants to investigate the cytological changes of axotomized sural nerves using histological, immunochemical, and quantitative image analysis combined with -omics approaches. Our examination of donor-matched intact and injured nerves revealed that the distal nerve segment experiences a major structural and cellular remodeling of all connective tissue layers within a 2-week time window post-axotomy. These changes occurred in concert with increased cellularization, vascularization, proliferation, and NGFR immunoreactivity, an early indicator of disrupted axonal support, in diverse cell types from the perineurial and epineurial sheaths. Whereas Schwann cells (SCs) did not expand in number, they clearly transformed their phenotype in response to the injury by becoming larger as they engulfed myelin debris and consistently -yet heterogeneously- increased NGFR expression and repair-associated genes. Nevertheless, most of the myelin content remained uncleared and ovoids were found in association with SCs rather than macrophages, which infiltrated poorly into the endoneurium at these early time points. Overall, our observations were consistent with a profile of slow Wallerian degeneration and modest SC activation overtaken by vascular development, ECM remodeling, and a strong reactivity of connective tissue cells. This is to our knowledge, the first description of early cytological changes in axotomized human nerves in an experimentally controlled injury paradigm.

## Introduction

The transplantation of nerve tissue grafts is a promising strategy to treat damaged circuits in the human brain [reviewed in [1]]. Our team has been among the first to surgically transect the sural nerve -a sensory nerve from the leg- to generate pre-conditioned nerve fascicles for autologous grafting in the brain in clinical trials for Parkinson’s disease (PD) [2, 3]. Participants of these trials, known as DBS-Plus, were implanted with segments of their own injury-activated sural nerve at the time of receiving Deep Brain Stimulation surgery [4, 5]. The clinical outcomes from the DBS-Plus trials have been favorable overall. A 2-year follow-up study reported no serious adverse events related to the grafted fascicles or the surgical intervention in a cohort of >18 transplanted participants [6]. Histological examination of post-mortem brain tissue from a handful of participants confirmed the long-term survival and integration of the nerve fascicles nearly 5 years after their implantation in the substantia nigra [7]. The promising histopathological and clinical outcome metrics encourage further testing of preconditioned (or activated) fascicle transplants for the treatment of PD, spinal cord injury, and other neurodegenerative conditions [8, 9].

The DBS-Plus trials were designed under the assumption that a transection injury would promptly activate mature myelinating and non-myelinating (Remak) Schwann cells (SCs), and induce their transition into a repair or reprogrammed state with enhanced pro-regenerative capabilities, as predicted from many years of research in animal models [reviewed in [10–12]]. Our earlier RNAseq and proteomics studies were consistent with this view, as they supported robust and consistent modifications in the sural nerve’s distal stump [1, 13], causing an enrichment in anti-apoptotic, mitogenic, and neurotrophic factors such as GDNF, NGF, and BDNF, among others. Even though our team has produced injury profiles through omics data, important questions regarding the dedifferentiation of human SCs and other cellular and molecular events triggered by the loss of axonal support -a process collectively known as Wallerian degeneration- remained unanswered.

This study took advantage of same-donor intact and transected nerve tissue samples and data retrieved from DBS-Plus participants to retrospectively understand how an axotomy changes the microscopic landscape of the adult human sural nerve. We found important commonalities in the cellular responses to injury in different trial participants, including solid morpho-histological, immunochemical, and molecular evidence on the initiation of the repair SC response, despite the divergence seen in individual cells and specimens, possibly as a consequence of donor variability. Nonetheless, it was obvious that axon and myelin removal was much less pronounced than expected from our understanding of injury responses in animal models. For instance, the upregulation of cJun, the archetypical marker of the SC repair phenotype [14], the infiltration of macrophages into the fascicles, and the induction of cell proliferation after injury were fairly modest, and the responses of individual SCs were heterogeneous. Whereas SCs did not substantially expand in number, they clearly transformed their phenotype as they became larger, engulfed myelin debris, and increased the expression of repair SC markers. Nevertheless, the overall regenerative response was slow or incomplete. Some axonal debris and most of the myelin content remained uncleared at 2 weeks post-injury, altogether suggesting that key events defining the process of Wallerian degeneration were slower than anticipated from observations in rodent models despite the massive transcriptional changes evidenced by RNAseq [1] and confirmed by proteomics analysis [13]. An unexpected response was the major structural and cellular reorganization in the distal nerve segment featuring widespread extracellular matrix (ECM) remodeling, vascular development, and cellularization of the connective tissue sheaths.

To conclude, our study contributes essential information to our collective understanding of the early microanatomy of the transected sural nerve. Our comprehensive depiction of the phenotype and state of differentiation of SCs and other nerve-derived cell types in homeostasis and repair in adult humans also highlighted important differences with animal responses. From a therapeutic perspective, these results attest to the value of performing a transection injury to increase the biological potency of peripheral nerve cells while providing a solid foundation for understanding and optimizing the use of preconditioned nerve tissue grafts in future investigations.

## Materials and Methods

### Reagents and antibodies

All primary antibodies were commercially available except for anti-human MPZ (Myelin Protein Zero) which was produced in our laboratory from hybridoma cell cultures (Bollesen et al. 1990), according to our established methods [15]. A detailed description of the source, type, specificity, and mode of use of the primary antibodies has been provided in Table 1. Secondary antibodies for immunostaining of formalin-fixed paraffin-embedded (FFPE) sections consisted of Discovery anti-mouse (Roche-Ventana, CAT: 760–4814) or anti-rabbit (Roche-Ventana, CAT 760–4815) HQ-conjugated antibodies, and Discovery anti-HQ-HRP (Roche-Ventana, CAT: 760–4820). Chromogenic detection of immunoreactive complexes on FFPE sections was done using 3,3’-Diaminobenzidine (DAB) peroxidase substrate (Roche-Ventana, CAT: 760–159). Other materials and reagents used for histology and immunohistochemistry (IHC) were the following: 10% Neutral Buffered Formalin (VWR, CAT: 89370–094), phosphate buffer saline 10X stock solution (PBS, Biorad), paraffin wax (Leica, CAT: 3801320), and permanent mounting reagent (Cytoseal XYL 8312–4).

### Nerve biospecimens

All biospecimens of intact and injured sural nerve were harvested as part of clinical trial NCT02369003, with a registration date 01/12/2015, examining the feasibility and tolerability of autologous nerve implantation in the brain of people with PD [5]. Nerve samples processed for FFPE were prepared from 4 participants (all males, with a mean age of 61 y/o, ranging from 48–71 y/o). Samples analyzed by RNA-seq were prepared from 6 participants (2 females and 4 males, between 53 to 70 y/o) [1]. Nerve samples analyzed by proteomics and immunoassays (ELISA) were procured from 14 (4 females and 10 males, between 52 and 70 y/o) and 15 participants (6 females and 9 males, between 51 and 69 y/o), respectively [13]. Only 3 out of the 15 samples used for ELISA overlapped with the ones used in proteomics analysis. The average time between surgeries (denervation time) was 14,83 and 14,21 days in samples used for RNAseq and proteomics, respectively. This study used surgical remains only. In all cases, the amount of tissue extracted from the participants was restricted to the minimum necessary for transplantation. Not causing unnecessary injury to the participants has been a strong guiding principle for the clinical work, despite limiting the amount of tissue becoming available for nonclinical work.

Two additional sural nerve samples used as intact-only controls were derived from tissue donors that had sustained an acute traumatic spinal cord injury (2 males, 40 and 70 y/o) and were enrolled in clinical trial NCT06243211 [17]. Given that these donors did not have PD, their nerves were used to represent the baseline condition for older adults. A banked traumatic neuroma specimen provided by the UK-Biospecimen Procurement & Translational Pathology Shared Resource Facility (BPTP-SRF) was used to test the immunoreactivity of MPZ and S100B antibodies (Table 1). The suitability of the biospecimens used for microscopy analysis was considered on a case-by-case basis after performing a thorough examination of H&E-stained sections to verify tissue integrity. Variables such as age, gender, and health conditions were recorded but not factored into the interpretation of results. The only condition factored into data interpretation was the time elapsed between the first and second surgery (see below), which was recorded as follows (14 days for donors-1 and −2, 12 days for donor-3, and 7 days for donor-4). Because the clinical trial design did not limit participants’ enrolment according to age, genetics, disease duration, or comorbidities, the existence of neuropathy or underlying conditions influencing nerve tissue regeneration cannot be ruled out. It should be mentioned that all transplanted participants who enrolled in clinical trial NCT02369003 had received a diagnosis of PD at least 3 years before trial enrollment. Nevertheless, immunohistochemical inspection of sural nerve biopsies (intact condition) revealed no obvious abnormalities or signs of neurodegeneration, i.e. axon or myelin loss. The impact of sample variability was investigated in a previous study that confirmed a strong degree of similarity within the intact and injured nerve groups based on calculating the Lin’s Concordance Correlation Coefficient [13]. The analysis of biospecimens and data in these studies was conducted after removing personal identifiers to prevent linkages to the identity of the donors.

### Sural nerve transection surgeries, nerve harvesting, and banking

Two sural nerve samples, denominated to as intact (or naïve) and injured (conditioned or activated) nerves, respectively, were collected from each participant of the NCT02369003 clinical trial. To harvest the intact nerve sample, the neurosurgeon identified the neurovascular bundle where the sural nerve was located in the ankle, and two sutures were tied around the nerve within 1 cm apart. A 1-cm segment of the nerve proximal to the sutures was then fully transected and excised without delay. Two weeks later (or as indicated in the figures), the ankle incision was reopened, suture markers were located, and a new 1–2 cm segment was collected from the distal nerve stump to serve as an implantable autologous fascicle graft. Segments dispositioned not required for implantation were fixed or frozen for lab work, as explained below. It is important to mention that the 2-step surgical design did not allow restoration of connections between the proximal and distal nerve stumps of the transected sural nerve within the time frame between the first and second surgeries. This setting rules out the possibility of axonal re-growth into the distal nerve segment.

For FFPE, biospecimens from the first and the second surgeries were placed in iced-cold sterile saline (PBS), transferred to cryovials, and fixed in 10% formalin for 48 h at 4 °C typically within 1–2 h post-harvesting. Samples destined for Omics were snap-frozen on dry ice immediately after harvesting and maintained at −80 °C until processing for analysis. The following publications contain more information on nerve tissue harvesting, storage, and analysis [1, 13]. Advances on the clinical progression of transplanted participants can be found in [2, 3, 6, 18–20].

### Nerve tissue embedding and sectioning

Paraffin embedding, sectioning, and immunostaining were done with the assistance of personnel from the UK-BPTP-SRF. In brief, formalin-fixed nerve tissues were placed inside Square Hole Tissue Processing Embedding Cassettes and immersed in a 70% ethanol solution for long-term storage. Next, these samples were embedded in paraffin wax at RT, sectioned into 4 μm-thick slices using a microtome, and directly placed onto Flex IHC glass microscope slides (Agilent, CAT: K802021–2) for secure adhesion. The tissue sections were stained immediately after sectioning, or kept at RT for not more than 1 week, to improve the reliability of immunostaining protocols.

### Histochemical stains and immunohistochemistry

All FFPE tissues were first assessed for their orientation and level of preservation using traditional Hematoxylin and Eosin (H&E) and Masson’s Trichrome green (TG) staining. Stained sections were evaluated carefully to understand the morphometry and general layout of the nerve tissues and to rule out obvious abnormalities. Collagen fibers are displayed as turquoise/green filamentous material, providing high contrast against red-stained structures and dark purple nuclei. For this reason, TG staining was particularly useful to reveal specific microstructures such as myelinated fibers and BVs in contrast to the collagenous ECM.

All IHC procedures were carried out using the Ventana Discovery Ultra with on-board deparaffinization according to the established protocols from the BPTP-SRF. Briefly, the sections were subjected to antigen retrieval using Ventana CC1 buffer and standard conditions except for Glut1 staining, which used Ventana CC1 mild antigen retrieval protocol. All antibodies were pre-diluted in buffer as suggested by the manufacturer (Roche) and incubated with the tissue sections at 37 °C for the times indicated in Table 1. The primary antibodies were then labelled with Discovery anti-mouse or anti-rabbit HQ-conjugated secondary antibodies for 20 min. This step was followed by an additional 20 min incubation with Discovery anti-HQ-HRP for staining with S100β, NGFR, Sox10, MPZ, NF-H, and SMA antibodies. OmniMap anti-rabbit-HRP was used to label Ki67 and Glut1 immunocomplexes. All slides were subjected to chromogenic detection using DAB substrate, counterstained in Mayer’s hematoxylin, and permanently mounted with Cytoseal reagent. The BPTP-SRF core provided anonymized positive control samples from their FFPE human tissue bank to confirm each and every round of immunostaining (see Table 1). A detailed description of histochemical and IHC protocols can be found in our recent work [16].

### Light and fluorescence microscopy and imaging

Bright field microscopy images of whole tissue sections were taken using a Zeiss AxioScan.Z7 slide scanner (available from the UK Light Microscopy core facility) or an Aperio AT2 Scanscope from Leica Biosystems (service provided by the Markesbery Neuropathology lab at the Sanders-Brown Center on Aging, UK). Imaging was performed using the following air lenses: 20X (NA=0.8, Plan Apo 20x/0.8 M27), 20X (NA= 0.75, Plan Apo), or 40X (with 2x automatic optical magnification changer). Fluorescence microscopy images of myelin autofluorescence under UV light were acquired with an Olympus XI71 microscope coupled to a color camera (6MP, 1” Sony HAD CCD sensor) with a 20X objective (NA=0.45). Bright field images of selected endoneurial areas stained with NGFR antibodies were overlaid with the respective UV fluorescence images to detect intact and degraded myelin profiles in relationship to NGFR immunoreactive cells. Microscopy images were analyzed to assess the contribution of selected components or markers of interest based on their distinctive localization, intensity, and/or distribution. Images from cross-sectional areas of donor-matched nerves were used for both qualitative and quantitative analysis. Longitudinal sections were used for qualitative analysis only, e.g., to observe profiles of myelin degradation.

Microscopy images were prepared for presentation using ImageJ (FIJI, NIH, open source) or Aperio Image Scope-Pathology slide viewing software. Images (RGB, 8-bit) were digitally processed only for the purpose of adjusting brightness and contrast in a homogenous manner using ImageJ software. The elements portrayed in the figures were organized for final display using Adobe Illustrator (Version 26.5).

### Quantitative image analysis

Microscopy image data were analyzed using semi-automated (ImageJ) and automated (HALO) software packages. ImageJ was preferred to combine automatic and manual counting of stained myelin and total nuclei because it offered a simpler option for the identification of selected regions of interest based on their location, shape, or color. Conversely, the AI-powered digital pathology software platform HALO (RRID:SCR_018350, Indica Labs, fee-for-license) was preferred for bulk analysis of nuclear counts (H&E) and DAB-stained areas in multiple serial sections. All microscopy images were imported with their corresponding metadata, and the default units were established in μm (or μm^2^) in all image datasets. Data retrieved from ImageJ and Halo analysis were imported into an Excel file for additional mathematical calculations. Images from donor-matched intact and injured tissues from at least 2 donors were processed for quantitative analysis. In most graphics, data from all fascicles (or other compartments) are shown individually to account for intra-specimen variability. All areas from a biospecimen’s section were included in the analysis except for those areas that had detached or overlapping tissue. Same-donor intact nerve samples that did not have large enough cross-sectional areas -or areas of sufficient quality- to serve as internal controls were not used for quantification but still shown as supportive qualitative data.

#### Morphometric measurements.

Image J was chosen to determine the general structural changes in response to injury by measuring cross-sectional areas of an entire nerve or its individual compartments. The surface areas of the whole nerve, the endoneurium, and the epi-perineurium were identified as per their typical structures, manually delimited using the freehand selection tool, and measured. The total endoneurial area was calculated as the sum of the areas of all fascicles combined. The connective tissue (CT) areas represented by the perineurial and epineurial areas combined were calculated by subtracting the endoneurial area from the total area. The ratio between each of these areas and that of the whole nerve, or other compartments, was calculated and plotted. A total of n=7 images from serial sections were analyzed per condition and participant to increase the analysis power. Areas with detached, distorted, or overlapping tissue were considered artifacts and were excluded from the image analysis in this and other measures.

#### Nuclear counts.

Manual counting (Image J) of DAB-stained endoneurial Sox10+ and perineurial Ki67+ nuclei was done to determine the relative content of SCs and proliferating perineural cells, respectively, as shown in [16]. Endoneurial and perineurial areas were delimited using the freehand selection tool, essentially as described above, and the nuclei were counted using the multi-point tool to estimate the percentage of Sox10+ and Ki67+ cells with respect to the total number of cells in each fascicle. Total cells were determined by the sum of hematoxylin-stained (blue) nuclei and DAB-stained (brown) nuclei. Alternatively, the Multiplex IHC algorithm (v3.4.9) was used to estimate total nuclear counts from hematoxylin-stained nuclei in endoneurial areas alone or together with Ki67+ or cJun+ nuclei. This script automatically discriminates and creates a mask of brown and/or blue nuclei within a region of interest (annotation layer) overlaid on the IHCimage. We optimized this algorithm for nerve tissue images by setting up the blue and brown color intensities and the fragmentation parameters as per the size of the nuclei in each layer of the nerve. To do so, a sampling area of each image was selected from intact and injured nerves using the Real Time Tuning analysis tool. Once the algorithm was optimized, annotation layers were delimited using the Pen tool to make each annotated layer correspond to the endoneurium of each fascicle. The area that corresponded to the intra-fascicular perineurium was excluded from the annotation layer by using the Scissor tool. Importantly, the analysis was performed after all annotated layers were identified. Snapshots of each annotated layer with or without the mask output were captured for graphical display. Results were exported to an Excel file to calculate the percentage of Ki67+ or cJun+ cells in reference to endoneurial cells per unit area.

#### Axonal and myelin content.

The size and density of NF+ and MPZ+ profiles were determined as a measure of axon and myelin distribution, respectively, using ImageJ. For axonal content, DAB+ areas were extracted using the Color Deconvolution 1.7 plugin package along with the H-DAB function, which splits the original RGB color image into three channels containing the blue, purple, and brown images, the latter corresponding to the NF signal. The scales were changed from pixels to μm since the scale from Color Deconvolution images is in pixels. The endoneurial areas were delimited for each fascicle using the freehand selection tool and duplicated to generate a mask of the positive staining using the Threshold plugin. Finally, the number and size of each axon were measured using the Analyze Particle plugin. A minimum particle size of 2 μm^2^ to infinity was set for NF+ areas to exclude particles that cannot be considered axons.

TG-stained sections were used as an alternative method for myelin quantification, as the images from intact and degraded myelin (reddish structures) can be separated digitally from other stained structures (such as vascular elements and collagen) with the image deconvolution plugin from ImageJ. A duplicated image (RGB format) of each fascicle was obtained, essentially by delimiting and measuring endoneurial areas as explained before, and the red color corresponding to myelin and BVs was isolated in each with the aid of the Color Deconvolution 1.7v plugin. The *User values* option was selected for each color, and RGB values were imported into the software. The image was split into three images, with Color 1 corresponding to the isolated myelin image, and the scale was changed from pixels to μm as explained above. A mask image from the deconvoluted one was created using the Threshold plugin, *Minimum* option, for each endoneurial area. To better detect intact myelin profiles, these were filled using the *Fill Holes* option from the Process-Binary menu. The Analyze Particle plugin was used to estimate the number and size of myelin figures per endoneurial area from each mask image. A minimum particle size of 10 um^2^ to infinity was set to exclude cell nuclei from the counting, and the circularity was set between 0.45 and 1.00 to avoid counting red staining derived from endoneurial elements other than myelin, including fibroblasts and BVs. Our combination of parameters excluded the cell nuclei and smaller myelin figures (<10um^2^). These parameters were optimized by using representative endoneurial areas of intact and injured nerve tissues before performing the analysis. The total number, average, and size distribution of axonal and myelin elements were expressed per unit of endoneurial area.

#### Perineurium thickness ratio.

The ratio between the perineurial area and the total fascicle area was calculated to determine the extent of morphological change in the injured perineurium, essentially as described in [21]. The freehand selection tool from ImageJ was used to discriminate the perineurium from the respective endoneurial areas in each fascicle. The outputs of these area measurements were imported into an Excel file for the calculation of the relative perineurial area in each fascicle. Various images from serial sections of the same biospecimen (n=4–7) were analyzed per condition and participant. Results are shown as the average measurement from each fascicle.

#### Analysis of the vasculature.

The blood vessels (BVs) were manually delimited using the freehand tool in ImageJ according to their typical structure, including the smooth muscle layer, regardless of their type or caliber. The BV areas were determined as the sum of all BVs located in the endoneurial or epineurial layers. To determine BV development in each compartment, we calculated the following parameters: (1) the ratio between the areas occupied by BVs and the total areas from the endoneurium and CT; (2) the number of BVs per unit area, and (3) the size distribution of the BVs per unit area. This analysis was conducted using images from serial sections (n=7) from each condition and participant.

#### Collagen content.

The amount and intensity of collagen staining in TG-stained sections were determined using an ImageJ methodology adapted from a published study [22]. The collagen content was used as a measure of ECM remodeling in the epineurium. Microscopy images (RGB format) were sampled randomly over the whole surface of the epineurium to select 10 – 12 square areas (250×250 μm) in each image for analysis. Because many of these areas were not entirely covered by tissue due to tissue detachment during washes and staining, the quantitative analysis was conducted using a mask of the image comprising tissue-containing areas only. The green color image (collagen) was isolated using the Color Deconvolution 1.7v plugin and the *User values* option, as explained above. A mask image from the isolated green channel was created using the Threshold plugin (d*efault* option) and used for measuring the green stain in regions of interest. The collagen content in each condition was estimated in reference to the effective epineurial areas rather than the total areas of the selected images to account for tissue loss.

#### Cytoplasmic and membrane stains.

The area quantification algorithm (v2.4.3) from Halo software was used to estimate the percentage of an area of interest that stained positive for a given marker (S100β, NGFR, and NF). The area script used was developed to discriminate DAB staining in sections counterstained with hematoxylin and creates a mask of DAB+ areas on top of the original image. The algorithm was optimized to differentiate DAB-stained areas with the Real Time Tuning analysis tool. Annotated layers were delimited according to the localization of the marker of interest and analyzed together. Snapshots from each layer were obtained as explained above. The output raw data, which included the total area and the area that was positive for each marker for each annotated layer (all expressed in μm^2^), was exported as an Excel file and used to make the final calculations.

### Bioinformatics analysis

RNA-seq [1] and TMT-MS proteomics [13] datasets from donor-matched intact (naïve) and injured nerve samples were reanalyzed to compare the expression of genes and proteins of interest out of 15,479 curated transcripts and 5,573 proteins (see Biospecimens). To comprehensively evaluate how gene and protein expression levels change after an injury, RNA-seq and proteomics results were examined in concert. The average values of the Log_2_ FC (fold change), indicating the ratio between the intact and injured condition in the identified genes/proteins (collectively referred to as features), were used as input data in a broad correlation analysis to understand the extent to which individual transcripts and proteins changed after injury. A total of 4,936 transcripts that had matching reported proteins, we calculated the ratio between injured and naïve samples out of the mean values of cyclic loess normalized MS3 reporter ion intensities and matched them with the corresponding ratio obtained from the mean gene expression levels (CPM) in the RNA-seq dataset. JMP Pro v. 17.2.0 statistical software was used for data visualization and calculation of linear regression between RNA-seq and TMT-MS fold change values (log_2_ scaled). Hierarchical clustering based on the Ward’s method was used to build a heatmap of differentially expressed genes/proteins in specific categories.

Available RNAseq data from our group [23, 24] and others [25–31] were used to select features representing the four main groups of nerve-resident cells, namely glial cells (SCs), connective tissue cells (also referred to as nonglial cells), immune cells, and vascular cells. To facilitate data visualization and interpretation, the features were organized according to their cell type specificity and function. SC-related genes (and their respective proteins, if identified) were subdivided as: (1) genes expressed in all lineage-committed SCs regardless of their stage of maturation (e.g. S100B, ErbB3, CDH19, Sox10); (2) genes expressed only in mature Remak SCs (CADM1, CDH2, L1CAM, NCAM1); (3) genes encoding for myelinating SC-specific transcription factors (EGR2 or POU3F1), adhesion molecules (CDH1), structural myelin proteins (e.g. MPZ, MBP, PMP22), proteins from the nodes of Ranvier (e.g. GLDN, CNTN1, CNTN2, NFASC), and enzymes linked to myelin lipid metabolism (e.g. UGT8, MAL, FABP7); and (4) genes expressed in repair (injury-activated) SCs (e.g. NEUM, JUN, FOS, NGFR, Sox2). Nonglial cell genes were organized into perineurial cell genes (e.g., SLC2A1, CLDN1, TJP1) and fibroblast genes (e.g., Thy1, CD34, Sox9). Immune cell genes were allocated according to their cell type specificity, as follows (1) macrophage genes (e. g. CD80, CCR2, MRC1); (2) neutrophil genes (e.g. CCL3, CCL19); (3) dendritic cell genes (e.g. CLEC4C, CLEC10A); (4) T cell & natural killer cell genes (e.g. IL7R, CCR7); and (5) B cell genes (CD79A, IGHM). Finally, genes expressed in vascular elements included: (1) smooth muscle genes (e.g. CNN1 or MYH11), (2) pericyte genes (FABP4), (3) genes expressed both in smooth muscle and pericytes (ACTA2, TAGLN), and (4) genes expressed in endothelial cells (e.g. GJA5, PLVAP) from veins (HIF1A or IL1R1) and arteries (IGFBP2, ITGA1, or ITGA5). For reference, Supplementary Table 1 includes the complete list of genes (n=290) and their corresponding identified proteins (n=137), organized according to the above-referred cell type specificity. To facilitate data visualization and identify changes in the expression levels of genes/proteins of interest in each participant, heatmaps were created using the Log_2_ FC (n=6 for RNAseq and n=14 for TMT-MS). The normalized counts (CPM) and the Log_2_ MS3 for RNAseq and proteomics data, respectively, were also plotted to denote the changing levels of expression of individual features representative of each category.

### Statistical Analysis

Graphical representation and statistical analysis of image data were performed using GraphPad Prism 9. Data are expressed as mean + SEM in the graphics. Statistical significance among treatments or conditions from microscopy image analysis was determined by unpaired parametric T-test (two-tailed). Significantly up- or down-regulated genes/proteins from the omics datasets were determined by the Holm-Šídák multiple unpaired T test method. P values <0.05 were considered statistically significant and are expressed as per their degree of significance according to GraphPad style: *p<0.05, **p<0.01, ***p<0.001, and ****p< 0.0001. A simple linear regression analysis was performed to establish if there was a relationship between the fascicle size and the number of cells using GraphPad Prism. The R-squared (R^2^) value or coefficient of determination is shown to indicate the correlation strength.

## Results

### 1. Analysis of intact and injured nerve tissues from clinical trial participants

**Figure 1:**
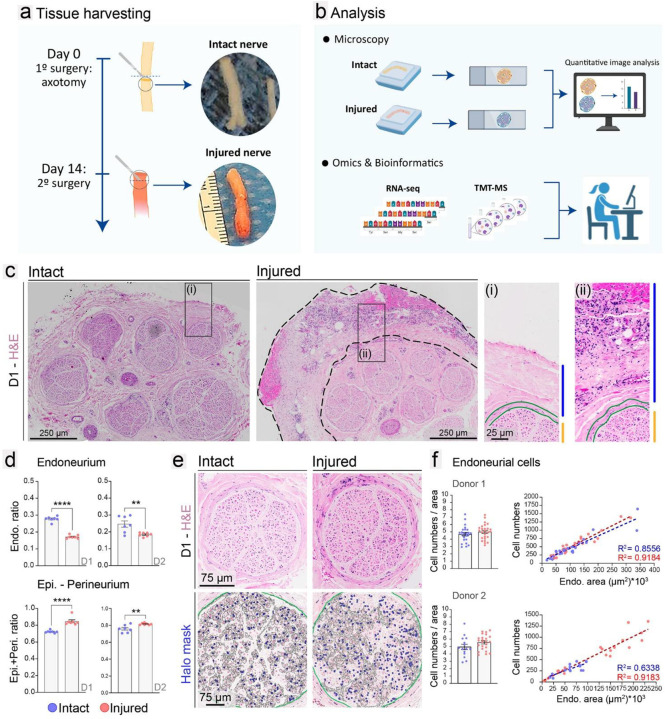
Procurement and analysis of intact and injured sural nerve tissues. **(a)** Our studies report data from sural nerve specimens collected at time zero (Intact nerve) and 2-weeks after a full transection procedure (injured nerve from the denervated stump). **(b)** Analysis consisted of histology and IHC followed by quantitative image analysis (upper panel) and bioinformatics of differential gene and protein expression profiles (lower panel). **(c)** Overall view of donor-matched sural nerve tissue (D1, donor 1) as seen in cross-section after staining with Hematoxylin and Eosin (H&E). Zoomed-in images of intact (i) and injured (ii) nerves are shown to depict structural features of the endoneurium (orange line), perineurium (green lines), and epineurium (blue lines). **(d)** Morphometric analysis evidenced connective tissue remodeling, including areas of new growth, concomitant with a relative reduction of endoneurial areas in injured nerves. Results are expressed as mean – SEM of n=7 independent tissue sections analyzed per condition and per donor. Each dot within the graphics represents the average value in each and all areas from independent sections in this and other graphs unless otherwise indicated. **(e, f)** Representative H&E-stained images and their corresponding HALO masks are shown in the upper and lower panels, respectively (e). Endoneurial cell density is expressed as the mean – SEM of n=3 independent sections, and each dot represents data from an individual fascicle (f, left panel). Linear regression analysis was performed to evaluate cell density changes with respect to endoneurial areas (f, right panel). The R^2^ values for each donor and condition are displayed in the graphics.

The availability of banked tissues from the DBS-Plus trials provided a unique opportunity to compare same-donor sural nerve samples before and after a transection injury. In most cases, the surgeons retrieved 2 nerve biospecimens from each participant, which were chemically fixed or snap-frozen as soon as possible to maintain tissue integrity. The *intact* nerve sample was obtained right after the surgeon performed a full axotomy on the sural nerve. The *injured* nerve sample was harvested from the corresponding distal stump two weeks (on average) after the initial incision (Fig. 1a). Gross examination of intact and injured nerve biopsies indicated that the axotomy induced robust changes observable by the naked eye, including thickening (or swelling) and redness across the length of the distal stump and especially within the areas adjacent to the transection site [1]. These observations prompted us to combine traditional and antibody-based histochemical stains to resolve the changes evoked by nerve damage. Microscopic observations of H&E-stained sections enabled us to identify general morphological and structural changes in defined histological layers or cell populations (Fig.1b) as well as guide the selection of cell-type- and stage-specific antibodies for immunostaining analysis. Finally, we extracted information from published RNAseq [1] and TMT-proteomics datasets [13] from a broader range of participants (Fig.1b) to both expand and support the microscopic observations.

### 2. Evidence of structural and organizational changes in injured nerves

Accumulated evidence has shown that nerve damage triggers a concerted pro-regenerative response in the distal nerve segment, which includes the conversion of mature SCs into repair cells capable of fostering nerve regrowth [reviewed in [32, 33]]. However, our current understanding of this process is based - almost entirely-on rodent models of nerve damage. Scarce information is available from adult humans except for chronic denervation injuries [34]. The availability of same-donor intact and injured nerve samples collected within a narrow time window post-axotomy allowed us to assess the early responses to injury in adult humans. Our histological observations indicated obvious changes in the overall landscape of the distal nerve stump entailing a remarkable structural reorganization of the connective tissue layers and the development of a heavily vascularized and densely populated epineurial layer (Fig.1c). A morphometric analysis of donor-matched biospecimens (2 donors) indicated an increase of epi-perineurial areas concomitant with a moderate reduction of endoneurial areas in the severed nerves (Fig.1d), consistent with earlier reports [35]. However, the multilayered perineurium, the fascicular organization, and the intra-fascicular content remained well-preserved (Fig.1e). A quantitative analysis of hematoxylin-stained nuclei confirmed that endoneurial cell density did not change after injury (Fig.1e, f) and was maintained constant irrespective of the caliber of the fascicles (Fig.1f).

These unexpected observations motivated us to perform a detailed immunohistochemical analysis to compare sections of intact and injured nerves using antibodies against markers for SCs (S100B, Sox10, NGFR), axons (NF), myelin (MPZ), perineurial cells (Glut1), macrophages (CD68), and the vasculature (SMA), (Table 1), essentially as reported previously [16]. Antibodies against cJun and the low affinity NGF receptor (or NGFR) were used to reveal injury-activated SCs. Anti-Ki67 and anti-CC3 were used to detect proliferating and apoptotic cells, respectively, and TG staining was used to differentially label collagen fibers. Because NGFR antibodies specifically labelled heterogeneous connective tissue cells [16], NGFR detection was useful to identify discrete populations of endoneurial and epineurial fibroblasts, and perineurial cells. Immunochemical changes were seen in all tissue layers. For this reason, we have structured the following sections to orderly explain the changes observed in the endoneurial, perineurial, and epineurial compartments. Whereas quantitative image analysis was used to characterize discrete cell types or elements in donor-matched samples, qualitative assessments were preferred for assessing non-donor matched specimens or establishing other comparisons. Importantly, we did not notice a distinctive immunological signature in the levels of expression and localization of our selected markers **(Sup. Fig. 1Aa)** or a diminished SC density **(Supp. Fig. 1b, c)** in intact nerves of PD participants when compared with non-PD controls.

### 3. Evidence of slow Wallerian degeneration after injury

**Figure 2:**
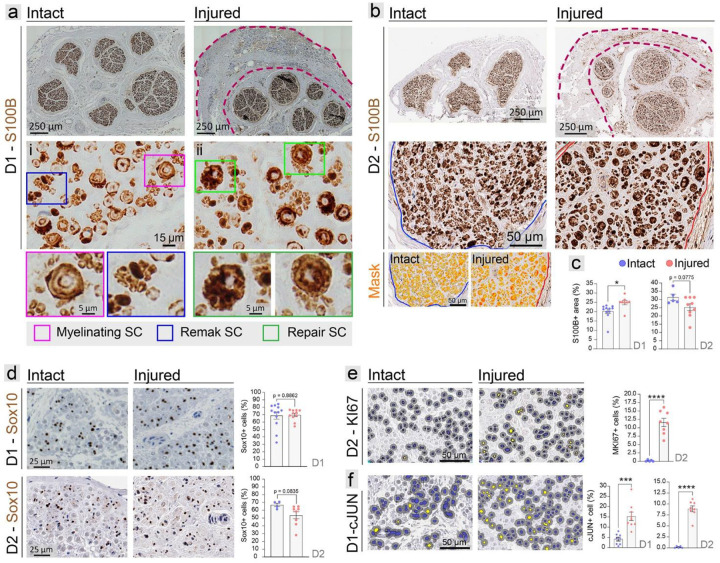
Evidence of Schwann cell activation after nerve injury. **(a-c)** Schwann cells (SCs) from intact and injured nerves stained with anti-S100B (brown). Myelinating SCs can be distinguished from non-myelinating (Remak) SCs based on their typical morphologies, as indicated in the selected examples (a, zoomed-in images). Repair SCs featured larger cell bodies in cross-section. Growth of epineurial tissue is indicated for reference in this and other images (red-dotted lines). Halo-assisted quantification of S100B staining is represented as the percentage of S100B+ cross-sectional areas in each fascicle. (c). Representative S100B+ areas and their respective masks (orange) are shown in (b), lower panel. **(d)** Sox10+ SCs (nuclear staining, brown) were quantified using ImageJ and represented as the percentage of Sox10+ cells with respect to the total endoneurial cells. **(e, f)** Halo-assisted quantification of Ki67 and cJun expression. Detection of Ki67+ and cJun+ nuclei (yellow dots, Halo mask) indicated a modest but significant increase in the proportion of proliferating/dedifferentiating cells (about 10%) in the endoneurium of injured nerves. Individual dots represent data from independent fascicles/areas in these (c-f) and other graphics.

To begin addressing how a transection injury affects the SC phenotype, we used antibodies against S100B and Sox10 to reveal all SCs from intact and injured nerves (Fig. 2a–d). Whereas the density of S100B+ areas and the percentage of Sox10+ nuclei were roughly the same (circa 70%) before and after injury (Fig. 2b–d), the size and morphology of individual SCs were deeply altered post-axotomy, as clearly visualized in S100B-stained sections and their respective HALO masks (Fig. 2a, b). While SCs seemed to remain confined within their basal lamina tubes, their cytoplasm became much larger, granular, and irregular after injury. Many SCs lost the morphology typical of myelinating and Remak SCs likely due to the loss of axon support and the engulfment of myelin and other debris into the SC’s cytoplasm (Fig. 2a). Altogether, we found that SC responses to injury were reflected mostly by an enlargement (hypertrophy) of the cells rather than an increase in SC numbers (Sox10+ nuclei), which aligns well with the maintenance of total endoneurial cellularity observed in H&E stains (Fig. 1f). Despite these observations, immunological detection and quantification of Ki-67+ nuclei provided clear evidence of injury-induced cell proliferation in about 10% of the endoneurial cells (Fig. 2e). Ki-67 was used here as generic indicator of cell cycle re-entry, as it is expected to be present in all phases of the cell cycle except for the quiescence state in the G_0_ phase [36]. A similar expression pattern was noticed for the AP1 transcription factor and immediate early gene cJun, as 10–15% of cells (on average) in the endoneurium of injured nerves displayed cJun+ nuclear immunostaining (Fig. 2f). We intended cJun to serve as repair SC marker based on extensive animal data linking cJun re-expression and the acute nerve injury response [10] despite its widespread role in the transduction and integration of mitogenic signals from a variety of tyrosine kinase and G protein-coupled receptors in many cell types [37]. Even though we were not able to confirm the precise identity of the endoneurial Ki67+ and cJun+ cells, these markers were induced modestly, which is in stark contrast with the strong induction of SC dedifferentiation and proliferation seen in rodents [38]. As expected, adult intact human nerves contained highly quiescent (Ki67-, cJun-) cells in all compartments except for the epineurium, where occasional proliferating (Ki67+) cells could be found in the resting state. Inspection of hematoxylin+ nuclei and CC3 staining did not provide evidence of apoptosis playing a major role in the injury response within the examined time window (Fig.1b).

**Figure 3:**
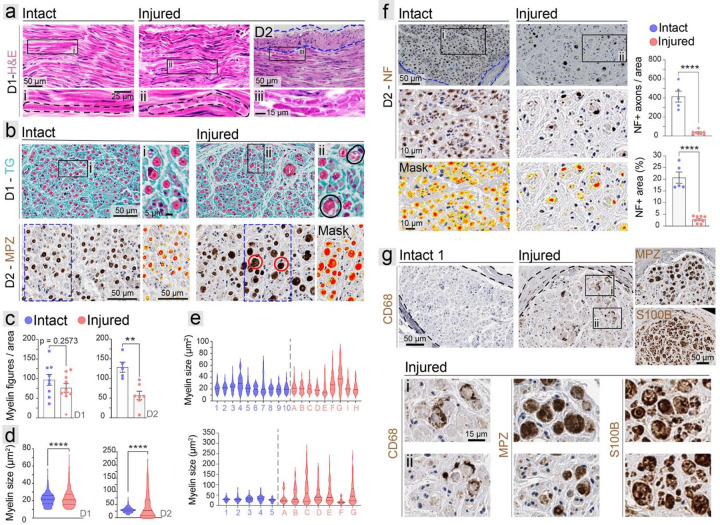
Evidence of slow myelin and axon degradation, and modest macrophage recruitment in injured nerves. **(a)** Representative longitudinal sections of H&E-stained intact (i) and injured fascicles featuring myelin ovoids (ii-iii, black, dotted lines) and epi-perineurial layer (D2, blue dotted lines). **(b)** Representative images of TG- and MPZ-stained tissue sections displaying fragmented myelin in the cytoplasm of SCs from injured nerves (black or red circles). **(c-e)** Quantitative analysis of myelin content (c), average size (d) and size distribution of myelin profiles in individual fascicles (e) from intact (blue) and injured (red) nerves. Each dot represents an individual fascicle in (c) and other graphics. **(f)** Quantitative analysis of NF expression in intact and injured nerves. Axonal degeneration was quantified as the number of NF+ particles (upper panel) and the percentage of NF+ endoneurial areas (lower panel). **(g)** Representative images of endoneurial areas stained with antibodies against CD68, MPZ, and S100B. Zoomed-in images (lower panels) feature CD68+ cells with a granulated cytoplasm in close proximity with myelin ovoids. Adjoined sections illustrate that most of the myelin debris are associated with S100B+ rather than CD68+ processes. DAB-stained tissue is shown next to the respective HALO masks in b and f.

A clear pattern of myelin breakdown was detected when injured fascicles were observed in longitudinal views (Fig. 3a). Despite these observations, quantification of myelin profiles from TG and MPZ-stained sections both indicated that the myelin content remained relatively unchanged in injured nerves (Fig. 3b, c). Higher magnification images of individual myelin figures evidenced the loss of the typical ring-like shape of intact myelin sheaths and the formation of ovoids of different sizes (Fig. 3b). Still, substantial variability in the degree of myelin fragmentation was observed from fascicle to fascicle and donor to donor. A quantitative analysis of particle size distribution clearly revealed an increase in the size of myelin profiles in injured fascicles, indicative of myelin degradation (Fig. 3d, e). Importantly, a comparison of adjacent tissue sections stained with MPZ and S100B antibodies further indicated that most MPZ+ profiles corresponded to S100B+ cells (Fig. 3g). These observations support the idea that SC enlargement after denervation results at least in part from the phagocytosis of myelin membranes, a hallmark of SC activation [39].

High resolution images and quantitative analysis of neurofilament (NF) immunostaining revealed that axonal elements were significantly reduced in transected nerves (Fig. 3f). Yet it was surprising to observe that a proportion of NF+ fragments remained uncleared at 2 w post-injury and that some axonal debris displayed intracellular cytoplasmic localization (Fig. 3f). Even though the density of macrophages increased in injured nerves, their representation in the injured endoneurium was modest (Fig. 3g), as judged by staining with antibodies recognizing CD68 (macrosialin), a glycoprotein commonly used as general marker for mononuclear phagocytes [40]. A proportion of CD68+ cells contained a granulated cytoplasm filled with debris. However, by comparing adjoined serial sections, it seemed clear that most myelin-loaded (MPZ+) cells expressed S100B rather than CD68 (Fig. 3g). Given that CD68+ cells were nearly absent in the intact endoneurium, we conclude that they could have infiltrated from the bloodstream, as suggested by reports linking CD68+ immunostaining and elicited macrophage infiltration after nerve injury [41]. Nevertheless, the image data suggest that macrophages contribute marginally to myelin uptake and degradation and that this function is accomplished primarily by SCs at least during the initial phase [5]. Variable responses were observed in the effectiveness of myelin and axon removal and the degree of macrophage infiltration, even when comparing individual fascicles from a single nerve. These results align well with reports showing slow and variable degeneration of peripheral axons in adult humans [34]. The time course of myelin and axon degradation in adult human nerves is unknown. Yet, our image data suggests that no relevant changes in axon and myelin degeneration occur within the first week post-injury **(Supp. Fig. 2)**.

### 4. Evidence of SC activation after injury and their conversion into repair cells

**Figure 4:**
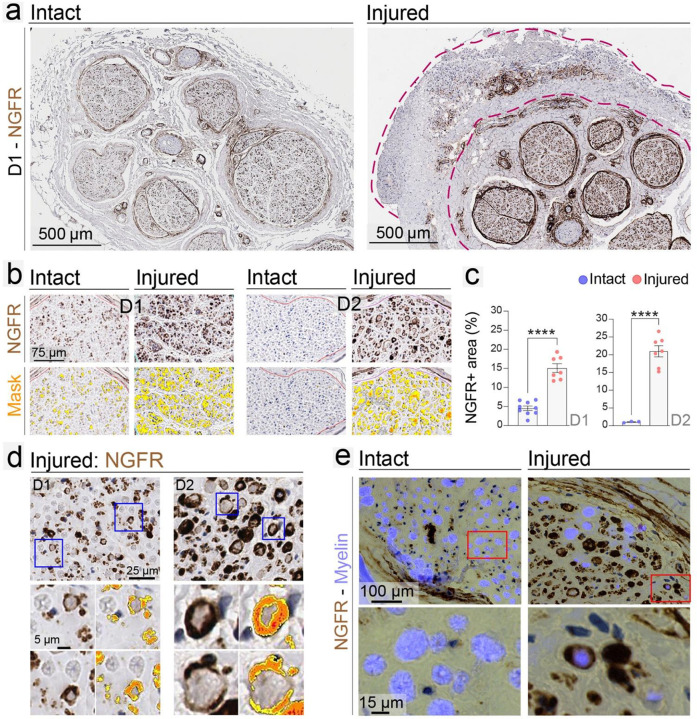
Evidence of SC activation in injured nerves. **(a)** Representative images of NGFR staining (brown) in intact and injured nerves provided evidence of NGFR induction in repair SCs and connective tissue cells. **(b, c)** Quantitative Halo analysis of endoneurial NGFR expression represented as the percentage of NGFR+ areas for all fascicles. **(d, e)** Representative images of injured nerves showcasing the expression pattern for NGFR in repair SCs (d). Endogenous myelin autofluorescence (blue) overlapped with the brown NGFR+ signal (e). Injury-induced NGFR+ cells were found to be both associated and not associated with myelin fragments.

NGFR upregulation has long been associated with peripheral glia activation in response to trauma and neurodegenerative disease [42–44]. As confirmed by quantitative image analysis, NGFR expression was robustly increased post-axotomy not only in (repair) SCs (Fig. 4a–c and **Supp. Fig. 3a)** but also in cells from connective tissue layers (Sections 5–6). Indeed, injured nerves characteristically exhibited heterogeneous NGFR+ cells, including myelin-associated SCs displaying an enlarged hypertrophic morphology that clearly differed from NGFR+ Remak SCs from intact nerves (Fig. 4d, e). High levels of endoneurial NGFR expression were observed in samples collected at 12 and 14 days rather than 7 days post-axotomy **(Supp. Fig. 3b)**. By shining UV light onto NGFR-stained sections, it was possible to observe endogenous myelin fluorescence in concert with the brown NGFR+ signal. We interpreted the finding of myelin ovoids within NGFR+ SCs as strong evidence for the SC’s conversion into repair SCs, considering that mature myelinating SCs do not express NGFR (Fig. 4e) and Remak SCs are not associated with myelin. However, the responses of individual SCs were heterogeneous. Myelin fragments were found in different stages of degradation, and only a proportion of these fragments were associated with NGFR+ processes (Fig. 4d, e). In addition, not all SCs increased NGFR expression to the same extent in injured nerves, as reflected by the many myelin fragments found in relationship to S100B+ rather than NGFR+ cells **(Supp. Fig. 3b)**. To conclude, this dataset confirmed that adult human SCs maintained a capacity to get activated, engulf myelin debris, and become repair cells after injury despite the relatively slow time course of Wallerian degeneration.

### 5. Evidence of perineurial cell activation and proliferation after injury

**Figure 5:**
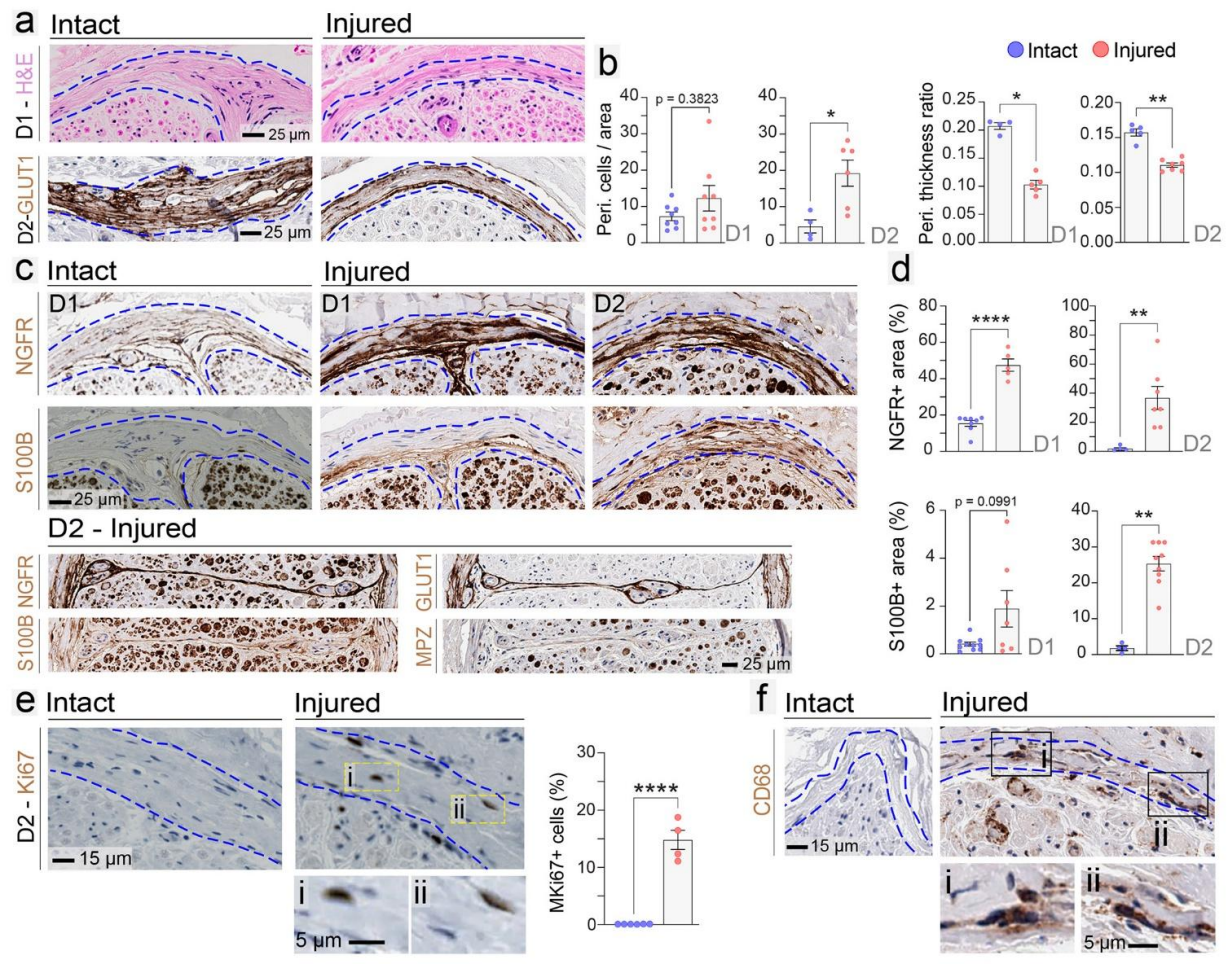
Evidence of perineurial cell activation after injury. **(a)** Representative images of the perineurial layer from H&E-stained (donor 1) and Glut1-stained (donor 2) intact and injured nerve fascicles. **(b)** Morphological changes included an increase in perineurial cell numbers (left) and a reduction in the thickness of the perineurium (right), as represented by the ratio between perineurial and the whole fascicle areas. **(c, d)** Representative images (c) and quantitative analysis (d) of the perineurium (upper panel), including the intra-fascicular sheath (lower panel), denoting an increase in NGFR and S100B expression after injury. **(e)** Representative images and quantitative analysis of nerves immune-stained with Ki67 antibodies, highlighting a modest but significant increase (about 15%) of Ki67+ cells in injured nerves. **(f)** Representative image of an injured nerve stained with CD68 antibodies showing CD68+ cells within the injured perineurium (insets).

Our results revealed that the human perineurium underwent a remarkable transformation involving major structural, cellular, and molecular changes, along with -and also mimicking- the SC’s conversion into repair cells. These changes included: (1) thinning of the perineurium with preservation of its typical concentrically arranged multilayered structure (Fig. 5a, b); (2) an increased cellular content as seen by total and proliferating perineurial cell nuclei (Fig. 5b, f); and (3) assorted immunochemical changes including a moderate reduction in the expression of Glut1, a glucose transporter that is selectively expressed in all perineurial cells, and a robust induction of NGFR in perineurial cell processes (Fig. 5c, d and **supp. Fig. 3c**). Quantitative analysis of hematoxylin-stained nuclei revealed that cell numbers increased in the injured perineurium (Fig. 5b) concomitantly with the appearance of cJun+ and Ki67+ nuclei (shown only for Ki67, Fig. 5f) specifically in perineurial cells. This interpretation is also consistent with the enhanced expression of NGFR in perineurial areas, including the intra-fascicular perineurial sheaths (Fig. 5c, lower panel). Even though perineurial NGFR levels were variable in intact nerves, NGFR expression after injury was strong and homogeneous, irrespective of the caliber of the fascicles (Fig. 4a), even at earlier time points **(Supp. Fig. 3a)**. Additional notable observations were perineurial cells becoming positive for S100B (Fig. 5c, d) without changing MPZ or Sox10 expression (shown only for MPZ), and the appearance of CD68+ processes (macrophages) in the injured perineurium (Fig. 5f), which mirrors the increase in CD68+ cells in endoneurial areas. It should be noted that, as opposed to NGFR, immunoreactive S100B+, Ki67+, and CD68+ cells are absent from the intact perineurium. We interpreted the increased cellularity, reduced Glut1, and increased NGFR expression in the perineurium to originate from the dedifferentiation and mitogenesis of perineurial cells. Although the early-stage remodeling with structural loosening of the injured perineurial barrier was an expected finding [45], the phenotypic conversion of perineurial cells emulating the SC’s conversion into repair cells was a novel finding.

### 6. Evidence of epineurium remodeling, cellularization, and vascularization after injury

**Figure 6:**
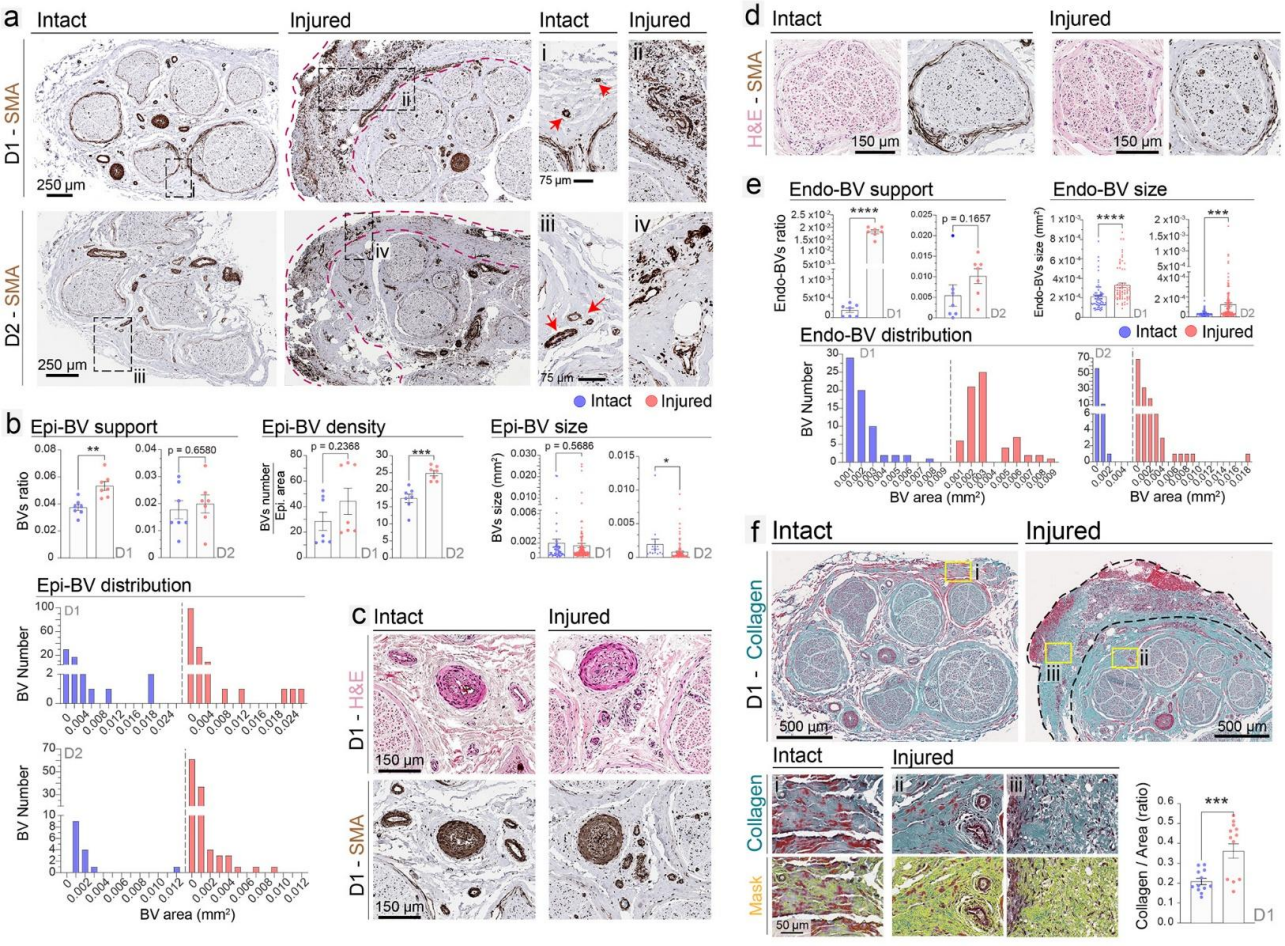
Vascular development and new growth of the epineurial layer after injury. **(a)** Low magnification images of SMA-immunostained nerve tissues before and after injury. The new epineurial growth -delimited by the red dotted lines in the injured nerves- contains an increased density of BVs (D1: i vs ii and D2: iii vs iv). **(c, d)** Representative images of corresponding H&E- and SMA-stained sections depicting increased vascular elements in the epineurium (c) and endoneurium (d). **(b, e)** BV development was determined by the proportional areas occupied by BVs (BV support), the number of BVs per unit area (BV density), the average size, and the size distribution of BVs in the epineurial (b) and endoneurial (e) layers. Results are expressed as the mean +SEM of n=7 tissue sections analyzed per condition and per donor. Each dot within the graphics represents the average value from independent sections. **(f)** Evidence of ECM remodeling. Collagen content (green color in TG-stained sections, yellow mask) was measured in n=12 ROIs (Regions Of Interest) from the intact and injured epineurium (D1 only).

As mentioned previously, the epineurium experienced dramatic structural and organizational changes post-axotomy that encompassed the development of a highly vascularized parenchymatous tissue that was most evidently appreciated in the outermost layers. The vascular changes were obvious in H&E- and TG-stained sections (Fig. 1c) but were most clearly revealed after staining with antibodies against SMA (Fig. 6a), a marker of myoepithelial and pericyte cells used here as a generic indicator of BVs. A morphometric analysis of donor-matched nerves revealed that injury-driven changes in the epineurial vasculature entailed an increase in BV density (Fig. 6a, b), primarily of smaller caliber vessels (Fig. 6c), which was indicative of neovascularization. Coincidentally, vascular density increased in the endoneurium of injured nerves, though the change in size distribution was less evident than in the epineurial layer (Fig. 6d, e).

**Figure 7:**
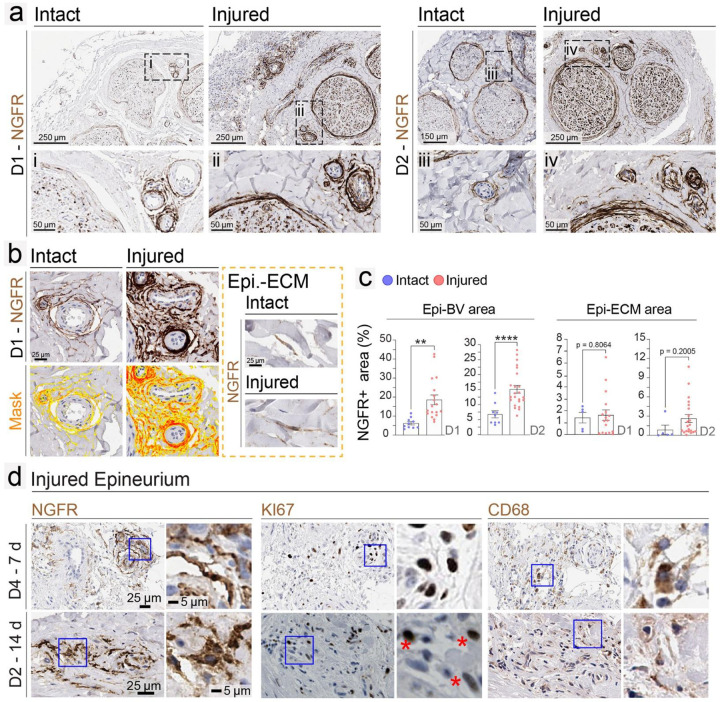
Evidence of epineurial cell activation in injured nerves. **(a)** Low magnification images of intact and injured sural nerves denote a strong induction of NGFR expression in epineurial fibroblast-like cells (Zoomed-in images). **(b, c)** High magnification images (b) and Halo-based quantitative analysis (c) of epineurial areas show a localized induction of NGFR in cells surrounding BVs but not in other areas (Epi-ECM). This analysis was performed in ROIs selected across the entire epineurium using n=10–18 (D1) and n=9–23 (D2) sections (Epi-BV area), and n=5–17 (D1) and n=5–21 (D2) sections (Epi-ECM area). **(d)** Injured nerve samples harvested at 7 and 14 days post-axotomy were immunostained for NGFR, Ki67, and CD68. Representative areas from adjoined sections are shown at low and high magnification.

The epineurium became stratified and heavily populated, particularly in those areas containing BVs. This was most clearly seen within the outer collagen-rich fibrotic tissue, where new growth occurs, shown as dense green color areas in TG-stained sections (Fig. 6f). Yet, collagen biosynthesis and BV development occurred across the entire epineurial surface. The epineurial layer featured a highly granular parenchyma (stroma) containing abundant fibroblast-like cells, a proportion of which were associated with BVs in various stages of maturation (Fig. 6a, c). Of particular interest was to observe NGFR immunoreactive cells surrounding pre-existing and newly formed BVs within the epifascicular and the interfascicular epineurial stroma (Fig. 7a, b, and **Supp. Fig. 3c**). In fact, most of the epineurial NGFR immunoreactivity was related to BVs, as the density of NGFR+ cells did not change in non-vascularized areas (Fig. 7b, c). Although epineurial NGFR immunoreactivity was detectable in intact nerves and variability was seen from region-to-region and sample-to-sample, the increase in NGFR expression around BVs was consistent across different donors, even at earlier time points (7 and 12 days) post-axotomy (Fig. 7d and **Supp. Fig. 3c**). It is possible that the granulated and heavily cellularized epineurial tissue originates from the concerted migration and proliferation of nerve-resident and infiltrating cells, as it seems to contain a mixture of fibroblasts and chronic inflammatory cells. Ki67 immunostaining provided further evidence that active cell division occurs in a proportion of stromal cells (Fig. 7d) located in discrete epineurial areas enriched in CD68+ phagocytes. A notable observation was the presence of CD68+ phagocytes at earlier post-injury time points (Fig. 7d). Altogether, the available data seem to suggest that the SC responses to injury are delayed and possibly overshadowed by an early and robust reorganization of cells in connective tissue layers.

### 7. Omics evidence of vascular, immune, and connective tissue cell activation after injury

**Figure 8:**
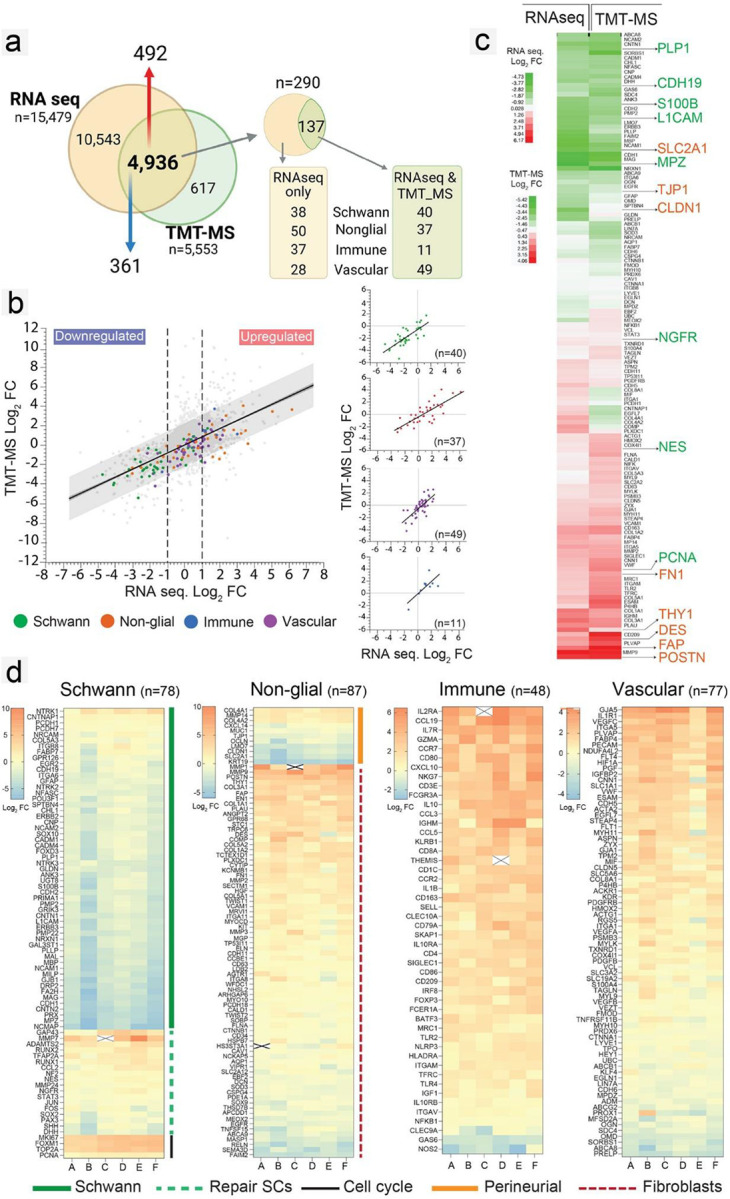
Omics evidence supporting a role for vascular, immune, and connective tissue cells rather than SCs to the injury response. **(a)** Bioinformatics workflow. The Venn diagram highlights the total and overlapping (4,936) transcripts/proteins (features) detected by RNAseq and proteomics analysis. The features that are up- and downregulated in both datasets (361 and 492, respectively) consisted of those exhibiting a |Log_2_ FC| > 1 (a 2X difference in expression). A discrete list of 290 transcripts, including 137 matching proteins representative of SCs, nonglial cells, immune, and vascular, cells was selected for further analysis. **(b)** A positive correlation between the overlapping genes and proteins was observed. Simple linear regression analysis is represented with a black line for all features [y=0.831*X + 0.02436] and features in each category, as follows. SCs [y=0.7553*X – 0.6334, R^2^=0.571], non-glial cells [y=0.6322*X – 0.4322, R^2^=0.629], vascular [y=0.9821*X - 0.573, R^2^=0.512], and immune [y=1.1018*X – 0.1863, R^2^=0.547]. **(c)** Heatmap representation of the average Log_2_ FC (average of all participants, intact vs injured) for features detected in both datasets. Representative SC (green) and nonglial cell (orange) features are highlighted in the heatmap for reference. **(d)** Heatmaps containing all selected features (RNAseq) per assigned category using data from the Log_2_ FC per participant. Subcategories of interest consisted of generic vs repair SC genes, perineurial vs fibroblast genes, and cell cycle genes, as indicated.

To look for further evidence in support of the abovementioned responses, we conducted a re-analysis of published RNAseq and TMT-MS datasets of intact and injured nerve samples from a larger group of participants (Fig. 8a). Our analysis highlighted a group of 4,936 transcripts (or proteins) present concomitantly in both datasets, which represented 32% and 88% of the total curated entries in the transcriptomics and proteomics datasets, respectively. We found that 361 downregulated and 492 upregulated genes/proteins out of the 4,936 overlapping transcripts/proteins exhibited a 2-fold difference in expression levels between intact and injured nerves (Fig. 8a).

To understand the nature of the cellular components involved in the injury responses, we looked into available human datasets from nerve tissues and isolated cells (see Materials and Methods section), and determined a discrete list of 290 genes specific for SCs (n=78), connective tissue cells, including fibroblasts and perineurial cells (nonglial cells, n=88), immune cells (n=48), and vascular cells (n=77) for independent analysis. To aid in omics data organization, the selected genes/proteins were in turn assigned to individual subcategories according to their representation in specific cell types or stages of maturation (**Supp. Table 1** and Fig. 8d). A correlation analysis unraveled consistent changes (i.e, upregulation or downregulation in both datasets) in the majority of the differentially expressed genes/proteins (Fig. 8b), including those selected for individual follow up (Fig. 8b, **insets**). The concerted shift in the RNA and protein expression profiles after axotomy was also evidenced when the genes/proteins of interest were represented in heat-map form either collectively (Fig. 8c) or in individual categories (Fig. 8d). Whereas most SC-specific features, including generic and myelinating SC-related genes/proteins, were down-regulated after injury, those representing non-glial, immune, and vascular cells were mostly up-regulated (Fig. 8b, d).

**Figure 9:**
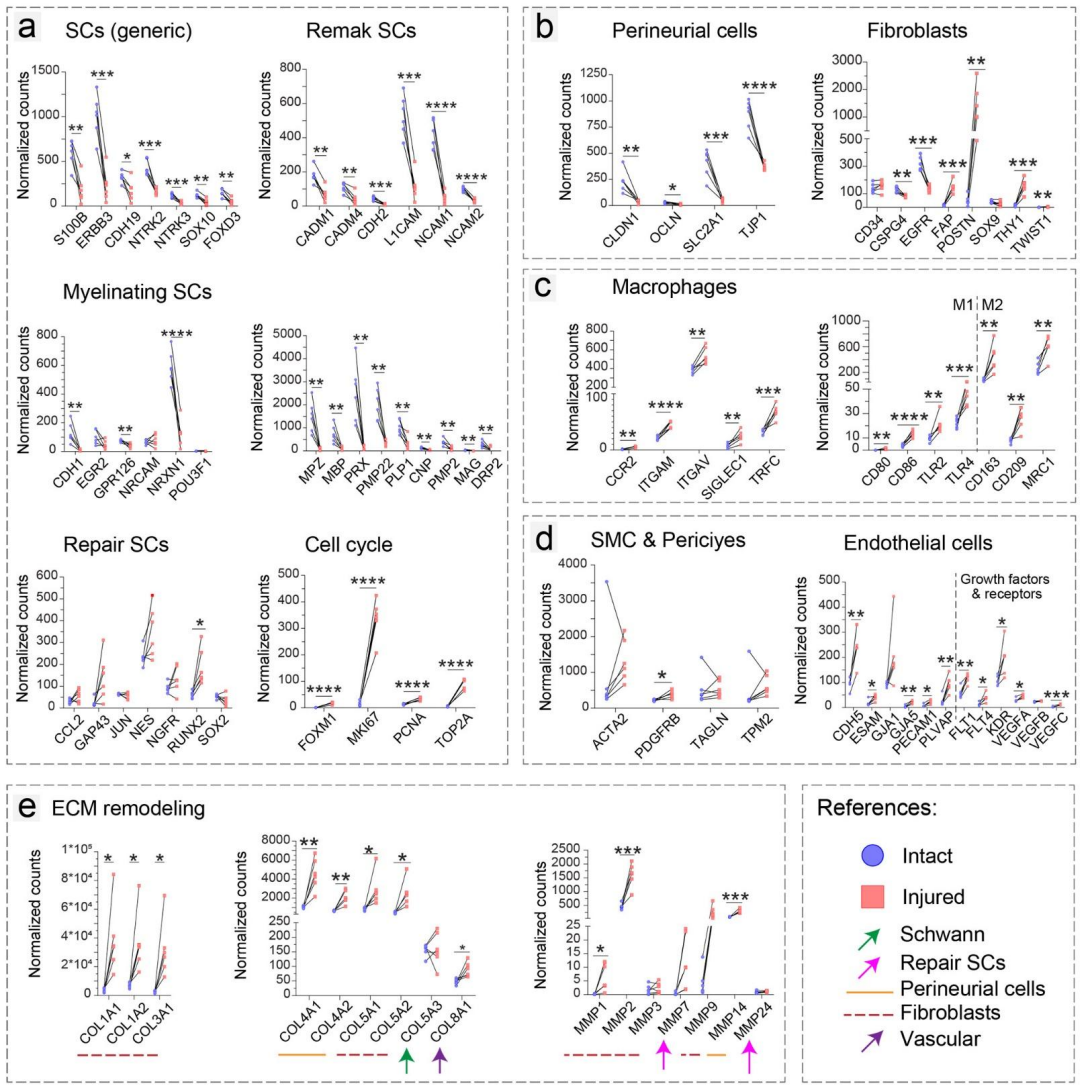
Injury-dependent changes in the expression levels of selected transcripts typical of SC, vascular, immune, and connective tissue cells. **(a-f)** Plots depict the gene expression levels (as normalized counts, CPM) per clinical trial participant (n=6). Representative genes from each category [i.e., SCs (a), nonglial cells (b), immune cells (c), vascular cells (d), and ECM genes (e)] were selected for display. SC genes were subdivided according to their representation in all cells from the SC lineage (e.g. S100B, Sox10), Remak SCs (e.g. CADM1, L1CAM), myelinating SCs (e.g. MPZ, PMP22), and repair SCs (e.g., NGFR, RUNX2). Genes representative of proliferating/cycling cells (e.g. PCNA and TOP2A) are shown in (A). Genes encoding collagen isoforms (COL) and ECM remodeling enzymes from the MMP family are shown separately (E), irrespective of their cellular localization. COL and MMP variants specific to a given type of cell or maturation state have been highlighted in color.

To identify changes in the gene/protein levels in each and all clinical trial participants, heatmaps (Figs. 8d and **Supp. Fig. 4**) were created using the Log2 FC values between injured and intact conditions for each of the 290 transcripts mentioned above, as well as their respective 137 matching proteins, segregated according to cell type-specific categories **(Supp. Table 1)**. Overall, proteomics results validated those of the RNAseq **(Supp. Fig. 4)**, although a protein signature was not detected for an important proportion of the sequenced transcripts, possibly due to a sensitivity issue. The responses to injury were consistent in samples from different donors, albeit rare exceptions, in both the transcriptomes and proteomes. Injury caused a downregulation of SC-specific genes/proteins except for some key repair SC genes that were modestly, yet consistently, up-regulated at the transcript and protein levels (Figs. 8–9 and **Supp. Fig. 4**). Importantly, the injury promoted a significant increase in proliferation-related genes such as MKi67 and PCNA in all participants (Figs. 8 and 9a), which aligned closely with the immuno-detection of Ki67+ cells in injured nerves only. Noticeably, the expression of JUN and SOX2 transcripts was low after injury, and the levels of NGFR were variable, possibly reflecting donor-to-donor and fascicle-to-fascicle variability.

Regarding connective tissue cells, a distinction could be made between genes associated with perineurial cell identity, which were mostly down-regulated (e.g. SLC2A1 and TJP1), and genes expressed broadly by fibroblasts, which were mostly up-regulated after injury (Figs. 8d, 9b and **supp. Fig. 4**). Fibroblast genes of interest encoded for key membrane glycoproteins (e.g., Thy1), cytoskeletal proteins (FAP), ECM proteins (POSTN) and enzymes involved in ECM remodeling (MMP1–3) previously used by our team and others to identify specific populations in nerve tissues [16]. Immune cell-related genes were among those showing the most drastic upregulation after injury, regardless of their cell type specificity, as these included specific genes typical of macrophages, neutrophils, dendritic cells, T cells & natural killer cells, and B cells (Figs. 8d, 9c, **Supp. Table 1**). Genes expressed in vascular elements were mostly up-regulated, including those from smooth muscle cells, pericytes, and endothelial cells (Figs. 8d and 9d). Genes common to both smooth muscle cells and pericytes, like ACTA2 (SMA protein) or PDGFRB, were also upregulated after injury, consistent with our immunohistochemical data. On a particular note, we found that the ECM remodeling enzymes of the MMP family and various collagen (COL) variants were strongly upregulated at both the gene and protein levels (Fig. 9e and **Supp. Fig. 3e**), indicating that a dynamic interplay between ECM breakdown and synthesis underlies the structural reorganization of the connective tissue. In addition, these changes align with our prior work reporting elevated protein and transcript levels of VEGF and neurotrophic growth factors e.g., BDNF and GDNF, after axotomy [13]. Available data from immunoassays [13] using samples obtained from the same clinical trial cohort is shown in concert with the respective transcript changes to show the overall correspondence between protein and RNA levels for secreted factors **(Supp. Fig. 5)**.

In sum, the omics data supported our immuno-histological findings and also highlighted the multifaceted events underlying the injury response. A major role of immunological (including but not restricted to macrophages), vascular, and connective tissue cells seems apparent. Whereas the downregulation of myelination-associated transcripts was consistent with SC dedifferentiation occurring at the transcriptional level, the upregulation of repair SC genes was less pronounced. Indeed, the contribution of SCs to the injury response seems either impaired or delayed when compared to the responses in other cell populations.

## Discussion

**Figure 10:**
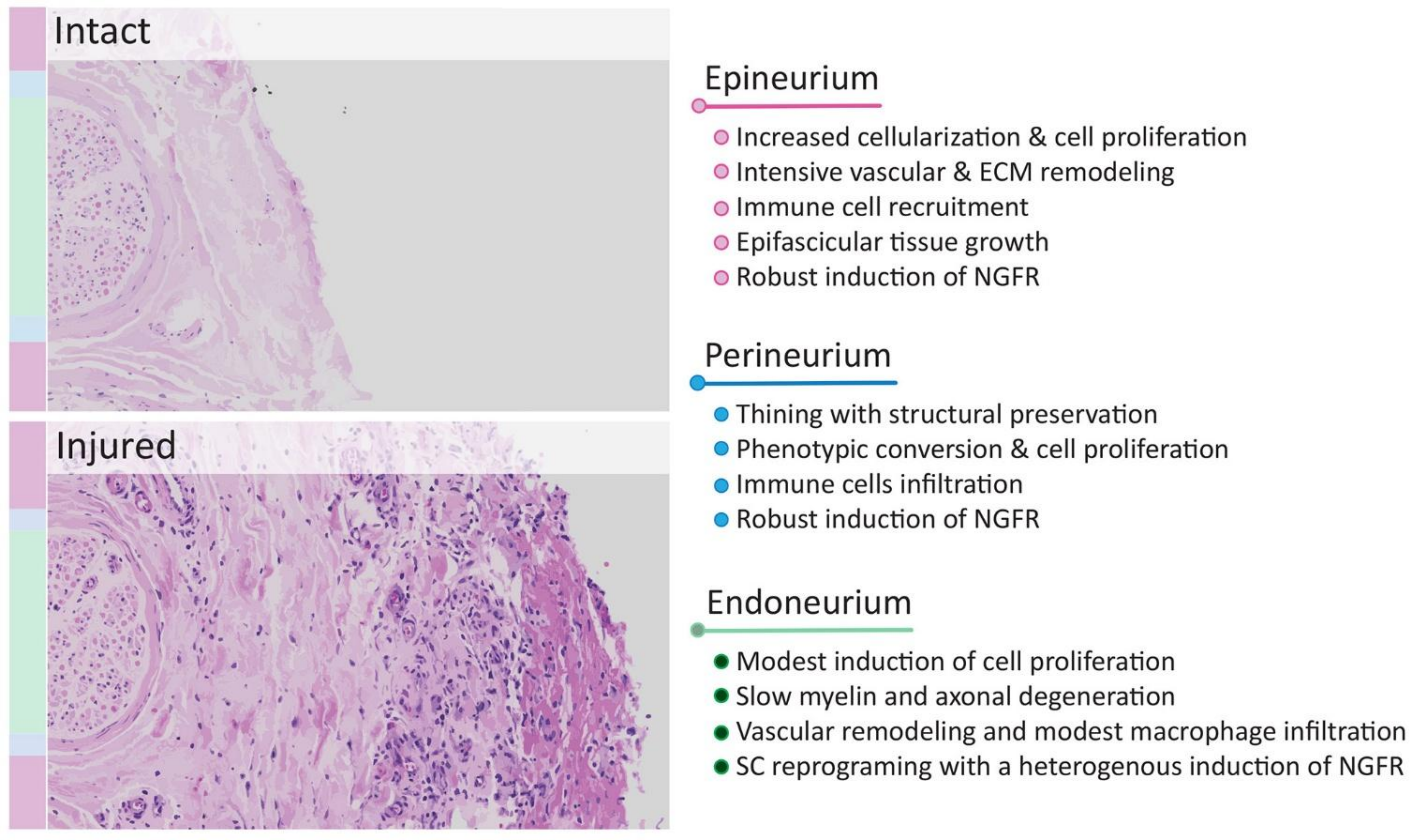
Cellular and molecular changes in the transected human sural nerve. The right panel describes the most prevalent injury-related responses subdivided by the tissue layer in which the changes occurred. Some events, such as increased cellular density, vascular development, increased NGFR expression, and cell cycle progression, are common to more than one tissue layer or type of cell. These data suggest a coordinated regenerative response involving cells from all connective tissue layers. The transformation of the vasa nervorum to allow for increased vascular supply and immune cell infiltration is perhaps the prime trigger for subsequent changes. The drawing on the left was created directly from the respective H&E images.

The goal of this study was to provide a nuanced and comprehensive understanding of the localization, structure, diversity, and state of maturation of cell types in the homeostatic and acutely injured human PNS. To reveal the most prevalent cellular and molecular responses to injury holistically (in all cells) and in situ (in each compartment), we relied on microscopic observations of representative specimens harvested before and after injury. We supported these findings by providing quantitative image analysis of critical constitutive elements from the endo, peri, and epineurium, and used omics data from a wider range of donors to validate and further inspect the results. Our studies indicated a complex and widespread transformation of the injured sural nerve occurring primarily within the connective tissue layers. Whereas SCs dedifferentiate and convert into myelin phagocytes, the responses associated with the onset and progression of Wallerian degeneration were both slow and heterogeneous. To our knowledge, the robust changes that occur within connective tissue cells have not been appreciated to this extent before. The main findings from our study have been summarized in Fig. 10.

### Cellular and molecular responses to a transection injury

A surprising finding was that the early responses to injury were not restricted to, and possibly not mainly driven by, SCs. We did not find evidence of expansion of the SC population, and most SC-specific transcripts/proteins were downregulated after injury, except for some repair SC genes. The appearance of Ki67+ nuclei in endoneurial cells, albeit at a low density, provided unequivocal evidence of cell cycle re-entry; however, many proliferating cells were found outside the endoneurium. This stays in stark contrast with experiments showing that almost all myelinating SCs undergo proliferation by 10 days in the transected mouse sciatic nerve [46]. We observed unambiguous changes in the morphology of SCs. Even though SC-mediated myelinophagy had begun by 2 w post-axotomy, most of the myelin debris remained uncleared within the cytoplasm of SCs, which became much larger and structurally disorganized. Axonal clearance was comparatively slow, as much axonal debris was seen as residual lumps within the cytoplasm of cells, likely SCs, based on S100B and MPZ profiling. A variable but still minor proportion of CD68+ macrophages was detected in proximity to SCs and myelin debris; yet, the contribution of macrophages seems insufficient for myelin clearance at this stage. Stierli et al (2018) reported that >22% of endoneurial cells in the transected murine sciatic nerve consisted of macrophages and that these accounted for 13% of all dividing cells at the peak of cell division that occurred as early as 4 d post-injury [46]. The appearance of myelin-filled NGFR+ cells was the strongest evidence supporting the repair SC phenotype [32]. It has been extensively documented that re-expression of the NGFR protein is a constitutive SC response to axonal degeneration [47, 48]. Yet, the heterogeneous levels of NGFR expression in denervated SCs indicate an asynchronous response that differs from the acute induction of NGFR in response to trauma [38, 46] and demyelinating insults in experimental animals [47]. The re-expression of NGFR and cJun in cells from the perineurium and the epineurial stroma further suggests enhanced reactivity in connective tissue cells, which matches the induction of NGFR in rat perineurial cells from severed nerves [49]. The recruitment of CD68+ cells to the epi-perineurium suggests their contribution to connective tissue remodeling and growth. The migration of epineurial macrophages through the perineurium has been proposed to be an alternative route for macrophage ingress into the endoneurial space based on the early (within 3 days) accumulation of macrophages in the epineurium before they appear in the endoneurium after rat sciatic nerve transection [50].

One notable finding was the hypertrophy of SCs in the distal stumps, which we have interpreted as morphological evidence of the mature-to-repair human SC transition. SC enlargement to form elongated cells with longitudinally aligned branched processes is one characteristic of SCs in Büngner bands [51]. Whether these morphological changes are linked to any given functional change needs to be explored further. Our analysis was insufficient to resolve whether any of the cycling (Ki67+) or cJun+ nuclei coincided with those of SCs. Proliferating cells and resident macrophages were rarely detected in uninjured sural nerve samples, as expected from the highly quiescent nature of mature nerves [46]. Thus, the detection of Ki67+, cJun+, and CD68+ cells after injury is a significant hallmark of change despite their modest representation (<15%) when compared to an analogous transection injury in mice, which is sufficient to drive dedifferentiation and proliferation in nearly all SCs within a 10-day time window [46].

How these responses to injury compare to those reported by others [reviewed in [11]] is still arguable. Establishing direct correlations between human and nonhuman data may be misleading, considering the longer human lifespan, genetic variability, and other factors. The slow rate of axonal growth and age- or disease-related impairments in SC support have been proposed to limit the efficiency of nerve regeneration in humans compared to animal models [52]. However, little information is available on the expected time course of changes in injured human nerves besides those reported by foundational literature [53, 54]. The handful of studies available to date have addressed chronic conditions spanning from months to years after trauma or the onset of neurodegenerative disease [34]. The early post-injury time points covered in this study, in relative time, have almost exclusively been explored in animal models. Nevertheless, it is worth noting that some of the events highlighted here are consistent with published data showing the relatively slow progression of human SC proliferation and cJun upregulation upon denervation, both peaking at around 90–100 days after a traumatic event [34]. The study by Wilcox et al. provided additional evidence showing that: (1) NGFR expression in SCs had increased as early as 10 days following denervation and that the upregulation of NGFR in denervated SCs was earlier than the upregulation of cJun; and (2) although NGFR mRNA levels were increased as soon as 4 d post-denervation, the induction ranged from a 10- to 100-fold through a time period of 170 days. Upregulation of NGFR protein and mRNA within 8 days was also observed when human nerves were pre-degenerated in vitro [55]; altogether, suggesting that NGFR upregulation is part of an early autonomous response to disrupted axonal support common to animals and humans [reviewed in [43]]. Even though NGFR is typically associated with dedifferentiating (denervated) SCs, its presence in perineurial and epineurial cells suggests that the molecular signals involved in NGFR induction may be shared across different cell types.

The accumulated data imply that the adaptive SC response to injury is not only faster and more robust in rodents than in humans but also shorter-lived in the former, as the repair SC phenotype is expected to be unstable and its survival eventually compromised in chronic injuries [56]. It is not clear whether human SCs develop and maintain a repair phenotype equivalent to the one observed in animals. The above-referred studies seem to suggest that the kinetics of SC activation and deactivation following an injury are both delayed in humans. NGFR upregulation seems to be long-lasting in human SCs, as evidenced by data showing that NGFR protein levels are maintained high levels for up to 100 d before declining to levels comparable to those of uninjured nerves [34]. It has been argued that SCs develop a fairly stable repair phenotype in human neuromas [57]. Isolated human SCs maintain high levels of NGFR and a repair-like phenotype almost indefinitely in vitro despite inductive signals for differentiation [58]. These observations raise the interesting possibility that the temporal window in which SCs sustain a repair state may be more extended in humans. Some evidence supporting this concept was provided by a morphological study of chronically transected human nerves carried out by Terenghi et al 1998 [59]. This study reported that S100B+ human SCs remained viable for at least 53 months within the distal stump of a severed nerve and that surviving SCs affected by chronic denervation were able to form typical bands of Büngner that closely resembled the ones described in animals based on their differential ultrastructural appearance and convoluted basal lamina [32]. Whether chronically denervated human SCs can maintain their plasticity to promote axon regrowth and/or re-differentiate into myelinating or Remak SCs is not known.

### A dominant role of connective tissue cells in the early injury response

Despite the seeming overlap in some of the initial molecular events, the question remains whether the responses to injury and repair are slower but mechanistically equivalent to those observed in rodents or the human responses involve a unique set of events and cellular players that do not have a direct rodent counterpart. The observation that a transection injury reduced the endoneurial compartment alongside, or possibly at the expense of, expanding the epineurium, is consistent with reports indicating endoneurial tube shrinkage and collagen deposition in the distal nerve stump [59]. Whereas the injured perineurium became thinner, it remained stratified and normal-looking rather than disorganized. A thinner perineurium may be easier to cross, which is supported by the observation of macrophage infiltration into the perineurial layer. The epineurial growth, though traditionally considered fibrotic scar tissue [45, 60], can be interpreted as the nerve’s attempt to create new bridging tissue to guide the reconnection of the proximal and distal stumps. The robust angiogenesis naturally develops to provide the necessary nutrients and oxygen and support the higher metabolic demands of tissue remodeling and growth in the nerve microenvironment [61]. These complex responses are orchestrated by various cell types, including interstitial fibroblasts, perineurial cells, vascular cells, and immune cells. Intriguingly, a cohort of diverse NGFR+ cells residing in the perineurial and epineurial sheaths [16] becomes activated, and possibly expands in number, in concert with connective tissue remodeling and neovascularization. Although strong perineurial NGFR expression has been observed in human neuroma biospecimens [57], reactive epineurial NGFR+ cells have not been previously reported in injured nerves. It has been known for some time that endoneurial fibroblasts interact with dedifferentiated SCs to guide regenerating axons into the distal stump during bridge formation [62] and proliferate alongside all other endoneurial cell types after a transection injury in mice [46]. It has also been reported that transcriptionally distinct mesenchyme-like progenitor cells from the rodent endoneurium contribute to non-neural tissue repair [63, 64]. It has been determined that the connective tissue ranges from 30% to 75% of a human nerve’s cross-sectional area according to the type of nerve, its location, the number of fascicles, and the proximity to joints [reviewed in [65]]. However, the contribution of human connective tissue cells to nerve injury and repair may have been underappreciated [65]. Our immunological profiling revealed highly heterogeneous fibroblast-like populations in all connective tissue layers [16], some of which display a strong pro-regenerative transcriptional profile and a mesenchymal phenotype after isolation and culturing [23]. Our findings agree with the understanding that the temporal evolution of the perineurium following an injury encompasses an initial phase wherein the tissue adopts a permissive, structurally plastic configuration, facilitating cellular infiltration that progressively transitions toward a re-established, reinforced barrier state (Hill & Williams 2003). Perineurial thickening is often associated with chronic neuropathic conditions or prolonged inflammatory states. However, the reactivity of human perineurial cells soon after injury, as evidenced by phenotypic and molecular markers and their capacity to proliferate again, was a novel finding that aligned well with evidence from zebrafish models [66].

### Implications for the development of biotherapeutic products

The notion that SCs can be transplanted to promote repair in the injured CNS and PNS is long-lasting [discussed in [67]]. Transplantation of cells generated in cell culture has been the gold standard in therapeutic translation of autologous SC therapies having reached the stage of clinical testing for spinal cord and nerve injuries. However, the compromised survival of isolated SCs shortly after transplantation remains a main roadblock for translating SC therapies in general [reviewed in [68, 69]]. Our team’s vast experience on the transplantation of undissociated nerve fascicles has consistently shown this modality to offer a more direct, expeditious, and cost-effective option for delivering SCs to a target organ such as the brain [discussed in [8]]. The delayed transplantation of a transected nerve enhances the bioactivity, and likely also the survivability of SC grafts while maintaining safety and compliance with accepted nerve transfer practices [8]. Activated fascicles are well-tolerated and the grafted SCs remain stable in the brain for many years [7], altogether supporting the benefits of the approach. The present studies reminded us that SCs do not work alone to orchestrate nerve tissue repair but also redirected our attention to the biotherapeutic potential of nerve-resident cells and infiltrating components. Whereas these findings reinforce the idea that a nerve transection is a safe and practical method to enhance the therapeutic value of transplantable nerve grafts, they also encourage rational refinements to the transplantation strategy, including extending the preconditioning time or selecting the fascicles used for implantation. Besides, our results can guide additional animal or in vitro work to evaluate experimental approaches that cannot be tested directly in humans, such as implementing non-surgical methods to lesioning the nerve tissue, as recently explored in Chu et al. (2025), [70]. We understand that the 2-stage surgical procedure used to generate activated nerve grafts in the DBS-Plus trials has not been attempted previously by other groups [70].

### Concluding remarks

These studies underscored an unexpected view of early-stage cellular dynamics in injured human nerves. With the material obtained from a handful of donors, we were able to detect some trends and consistencies that seem to occur irrespective of donor differences. Even though we have discussed our results in light of previous observations in experimental animals, the analysis of nonhuman nerves was discouraged to maintain the clinical relevance of the work. In fact, we have consistently found that the transcriptional and antigenic properties of rodent nerve cells, including SCs, do not faithfully represent the human condition [23, 58]. Here, we emphasized on direct microscopic observations to not only bypass critical interspecies variability but also provide a more accurate reflection of the histophysiology, cellular architecture, and immune responses that are unique to humans. Molecular (omics) data was useful to incorporate results from a more diverse and larger set of samples. Yet, we interpreted the findings from omics profiles with caution, as they cannot reflect the cytological events and cell-cell interactions typical of tissues under dynamic remodeling.

We believe this work provides a baseline for understanding nerve degeneration in adult humans, as the injury type, the anatomic location, the time post-injury, and other factors were kept under experimental control. Nevertheless, we do not intend to generalize our findings or imply the responses are typical, as nerve tissues can vary substantially in aging subjects [71]. Because our study is biospecimens-centered and the samples were collected independently from one another, we maintained strict quality control standards to validate the integrity of the samples and reduce pre-analytical variability. Our goal was to maximize data quality and biological significance while accounting for the small, potentially variable, and also irreplaceable, samples used. We acknowledge that these and other limitations, such as donor variability and the effect of aging [72] or PD [73], could have contributed to our results. Nevertheless, the broader and more immediate potential for clinical translation of our findings outweighs the limitations, as information retrieved solely on the basis of nonhuman models may fail to represent critical human biological events.

## Supplementary Material

Supplemental Information

This article includes one supplementary Table and five supplementary figures. **Supp. Fig. 1** contains microscopy images characterizing healthy nerves relevant to Fig. 1. **Supp. Figs. 2–3** show a temporal progression of axon and myelin degradation, and the expression of NGFR, which are relevant to Figs. 3–4, respectively. **Supp. Figs. 4–5** show data from immunoassay and RNAseq and are relevant to Figs. 8–9. **Supp. Table 1** contains the RNAseq and TMT-MS datasets used to perform the correlation and expression analysis displayed in Figs. 8–9 and **Supp. Figs. 4–5**.

Supplementary Files

This is a list of supplementary files associated with this preprint. Click to download.

- SupplementaryFigures03.23.2026.pdf
- AparicioetalSuppTable1.xlsx

## Figures and Tables

**Table 1:** Primary antibodies used for immunostaining. The table describes the source, type, mode of use, and specificity of the monoclonal (mAb) and polyclonal (poly.) antibodies used in this study. Banked human tissues were used to confirm the antibody specificity and reactivity in all immunoreaction rounds (right column). Unless specified, the antibodies were used as per established protocols from the UK-BPTP SRF. The working concentration for anti-S100B (*) was determined by testing positive control tissue within a dilution range of 1:100–1:20,000. Conditioned medium (CM) from cultured HB-592 (anti-MPZ) hybridoma cells (kind donation of Melitta Schachner) was used in the form of cleared undiluted cell culture supernatant. NGFR: Nerve Growth Factor Receptor. MPZ: Myelin Protein Zero. NF-H: Neurofilament, high chain. Glut1: Glucose transporter-1. SMA: Smooth Muscle Actin. CC3: Cleaved Caspase 3.

Antigen	Clone / HB	Species	Company	Catalog number	Dilution / Incubation time	Subcellular localization	Cell type specificity	Positive control tissue
**Sox10**	SP267	Rabbit IgG mAb	Roche - Ventana	760–4968	Prediluted / 1 h	Nuclear	SCs only	Skin
**S100β**	-	Rabbit poly.	Dako - Agilent	Z0311 - GA504	1:4,000 * / 1 h	Cytoplasmic (typical) and nuclear (atypical)	SCs Perineurial cells stain mildly only after injury	Tonsil
**NGFR**	MRQ-21	Mouse IgG1 mAb	Roche - Ventana	760–4391	Prediluted / 32 min	Membrane	Various cell types [16]	Breast cancer panel
**MPZ**	HB-592	Mouse IgG1 mAb	Produced in house		CM / 1 h	Membrane/Myelin	Myelinating SCs	Neuroma
**NF-H**	2F11	Mouse IgG1 mAb	Roche - Ventana	760–2661	Prediluted / 24 min	Cytoskeletal	Neuronal (axons)	Brain
**Glut1**	-	Rabbit poly.	Roche - Ventana	7660–4526	Prediluted/16 min	Membrane	Perineurial cells and red blood cells	Colon
**SMA**	1A4	Mouse IgG4 mAb	Roche - Ventana	760–2833	Predilute / 1 h	Cytoplasmic (typical) and nuclear	Pericytes, smooth muscle cells, and a subset of perineurial cells	Breast cancer
**CD68**	KP-1	Mouse IgG1 mAb	Roche – Ventana	790–2931	No dilution / 20 min	Membrane	Macrophages	Tonsil
**cJUN**	60A8	Rabbit mAb	Cell Signaling	9165L	1:200 / 1 h	Nuclear	Assorted cell types	Vagina
**MKi-67**	30–9	Rabbit mAb	Roche - Ventana	790–4286	1:100 / 1 h	Nuclear	Assorted cell types	Tonsil
**Sox2**	SP76	Rabbit mAb	Roche - Ventana	760–4621	Prediluted / 1 h	Nuclear	SCs only	Lung
**CC3**	ASP175	Rabbit mAb	Cell Signaling	9661	1:150 / 1 h	Nuclear	Assorted cell types	Tonsil

## Data Availability

RNAseq data that support Figs. 8 and 9 and Supp. **Fig. 5** are available in the online supplemental material of [1]. TMT-MS data that support Fig. 8 and **Supp. Fig. 4** are publicly available in UKnowledge Neuroscience Research Data repository at https://doi.org/10.13023/cbz6-ea76.

## Abbreviations

SCs: Schwann cells
BVs: Blood vessels
ECM: Extracellular matrix
Endo: Endoneurium
Epi.: Epineurium
Peri.: Perineurium
PD: Parkinson’s disease
FFPE: Formalin-Fixed Paraffin-Embedded
IHC: immunohistochemistry
H&E: Hematoxylin and Eosin stain
TG: Masson’s Trichrome green
